# Pediatric hypnic headache: a systematic review

**DOI:** 10.3389/fneur.2023.1254567

**Published:** 2023-08-11

**Authors:** Alessandro Ferretti, Margherita Velardi, Claudia Fanfoni, Giovanni Di Nardo, Melania Evangelisti, Thomas Foiadelli, Alessandro Orsini, Marco Del Pozzo, Gianluca Terrin, Umberto Raucci, Pasquale Striano, Pasquale Parisi

**Affiliations:** ^1^Pediatrics Unit, NESMOS Department, Faculty of Medicine and Psychology, Sapienza University, S. Andrea Hospital, Rome, Italy; ^2^General and Emergency Department, Bambino Gesù Children’s Hospital, Istituto di Ricerca e Cura a Carattere Scientifico, Rome, Italy; ^3^Pediatric Clinic, Fondazione IRCCS Policlinico San Matteo, Pavia, Italy; ^4^Pediatric Neurology, Pediatric University Department, Azienda Ospedaliera Universitaria Pisana, University of Pisa, Pisa, Italy; ^5^Department of Mother and Child, Gynecological and Urological Sciences, Faculty of Medicine and Dentistry, Sapienza University of Rome, Rome, Italy; ^6^Pediatric Neurology and Muscular Diseases Unit, IRCCS Istituto Giannina Gaslini, Genoa, Italy; ^7^Department of Neurosciences, Rehabilitation, Ophthalmology, Genetics, Maternal and Child Health, University of Genoa, Genoa, Italy

**Keywords:** hypnic headache, child, sleep, melatonin, systematic review, primary headache, international classification of headache disorders 3rd edition

## Abstract

**Introduction:**

Hypnic headache (HH) is a primary headache, and it is considered a rare condition in children. The underlying mechanisms of HH are not yet fully understood. This systematic review aims to provide a comprehensive description of the clinical features of all published cases of pediatric HH. It will also discuss the differences in headache features between children and adults, the increased diagnostic sensitivity of the new diagnostic criteria (ICHD-3), potential pathophysiological hypotheses explaining the higher incidence in adults, differential diagnoses, and therapeutic options for children.

**Methods:**

A systematic search was conducted to identify and analyze articles reporting cases of HH in patients under the age of 18. The search was performed in major medical databases including Cochrane Library, EBSCO, Embase, Medline, PubMed, Science Direct, Scopus, and Web of Science. The search covered the period from 1988 to April 2023. Relevant studies were screened for eligibility, and data extraction was performed using a standardized approach.

**Results:**

Seven children with HH were included in the analysis. The mean age of onset for headache attacks was 10 ± 4.3 years (range 3–15 years). The average time from the start of headaches to diagnosis was 15.8 ± 25.0 months (range 1–60 months). Headache features in children differed from those observed in adult HH patients. Children experienced throbbing/pulsating pain, while adults reported dull/pressure-like pain. Children also had lower frequency and shorter duration of attacks compared to adults. The use of ICHD-3 criteria appeared to be more sensitive and inclusive for diagnosing HH in children compared to the previous ICHD-2 criteria. The association of headache attacks with sleep suggests that HH may be a primary disorder with a chronobiological origin. Hypothalamic dysfunction and melatonin dysregulation, which are more prevalent in older individuals, could potentially explain the higher incidence of HH in adults. Other primary headaches and secondary causes should be ruled out. Melatonin prophylactic therapy may be considered for pediatric patients.

**Discussion:**

Further evaluation of the clinical features of HH in children is needed. The development of specific diagnostic criteria for pediatric cases could improve diagnostic rates and enhance the management of children with HH.

## 1. Introduction

Hypnic headache (HH), previously referred to as “alarm clock headache” or “clockwise headache” (1, 2), is a primary headache disorder predominantly observed in the elderly population (3, 4). Despite being relatively uncommon, approximately 350 cases have been documented in the literature since its initial description by Raskin in 1988 (5, 6). Although the exact pathophysiology of HH remains to be fully elucidated, it is presumed to involve a disturbance of chronobiological rhythms given its circadian nature. Dodick et al. (2) estimated a prevalence of 0.07% in children based on one HH diagnosis for every 1,400 headaches evaluated annually at the Mayo Clinic. In a specialized Spanish headache clinic, it was found that one out of every 100 children with strictly unilateral headaches experienced HH (7). A recent study in Iceland reported a prevalence of 0.22% for probable HH among 921 participants, highlighting the relative rarity of this condition in the pediatric population (8). As pediatric cases of HH are infrequent, the exact prevalence in this population has yet to be determined; however, an improved understanding of this disorder may lead to increased diagnoses across all age groups. Initially, Raskin’s description of patients indicated a clear male predominance (5). However, subsequent studies suggested a higher incidence among women (4, 6, 8–10). Diagnostic criteria for HH were first proposed in the International Classification of Headache Disorders 2nd edition (ICHD-2) in 2004 (11) and were subsequently revised in the ICHD-3 (12) (see Table 1). According to the latest criteria outlined in the ICHD-3 (12), HH is characterized by recurrent headache attacks that occur exclusively during sleep, leading to awakening and lasting up to 4 h. These attacks must occur on at least 10 days per month for a minimum of 3 months, with a duration ranging from 15 min to 4 h after awakening and should not present with any cranial autonomic symptoms or restlessness. Furthermore, the diagnosis of HH should be made in the absence of any other ICHD-3 diagnosis that may account for the symptoms (10, 12). The ICHD-3 also distinguishes between HH and probable HH (see Table 1), the latter being a more inclusive diagnosis. These criteria, introduced in the ICHD-3 beta, demonstrate greater sensitivity for identifying HH compared to the criteria in the ICHD-2 (9). The objective of this systematic review is to provide a comprehensive overview of the clinical features observed in all documented cases of pediatric HH to date. The review will explore the distinctions in headache characteristics between pediatric and adult populations, highlight the enhanced diagnostic sensitivity of ICHD-3 in pediatric patients compared to ICHD-2, discuss potential pathophysiological hypotheses underlying the increased incidence of HH in adults, explore differential diagnoses, and examine therapeutic options available specifically for children.

**Table 1:** Diagnostic criteria of hypnic headache and probable hypnic headache according to ICHD-2 and ICHD-3.

Hypnic headache			Probable hypnic headache	
ICHD-2 (code 4.5)		ICHD-3 (code 4.9)	ICHD-3 (code 4.9.1)	
Dull headache fulfilling criteria B–D	**A**	Recurrent headache attacks fulfilling criteria B–E	Recurrent headache attacks fulfilling criteria B and C	**A**
Develops only during sleep and awakens the patient	**B**	Developing only during sleep, and causing wakening	Developing only during sleep, and causing wakening	**B**
At least two of the following characteristics: occurs >15 times per month; lasts ≥15 min after waking; first occurs after age of 50 years	**C**	Occurring on ≥10 days/month for >3 months	Two only of the following:	**C**
No autonomic symptoms and no more than one of nausea, photophobia or phonophobia	**D**	Lasting from 15 min up to 4 h after waking	Occurring on ≥10 days/month for >3 months	**D**
Not attributed to another disorder	**E**	No cranial autonomic symptoms or restlessness	Lasting from 15 min up to 4 h after waking	**E**
	**F**	Not better accounted for by another ICHD-3 diagnosis	No cranial autonomic symptoms or restlessness	**F**
			Not fulfilling ICHD-3 criteria for any other headache disorder	**G**
			Not better accounted for by another ICHD-3 diagnosis	**H**

## 2. Materials and methods

We conducted a systematic search for patients with onset of hypnic headache (HH) under the age of 18, focusing on observational studies (prospective and retrospective cohort), case reports, and case series indexed in major medical databases (Cochrane Library, EBSCO, Embase, Medline, PubMed, Science Direct, Scopus, and Web of Science) published from 1988 (the year of the first description) to April 2023. Our search strategy involved the following terms: (1) “hypnic headache” and “child”; (2) “hypnic headache” and “adult”; (3) “hypnic headache” and “child or adult.” Additionally, we screened the references of the identified articles to identify any additional publications that met our research criteria. This systematic review adhered to the Preferred Reporting Items for Systematic Reviews and Meta-Analyses (PRISMA) guidelines (13). Data were extracted from the original articles using a standardized data collection form. The extracted information included the first author’s name, publication year, study design, demographic characteristics, HH features, diagnostic tests performed, therapeutic experiences, and clinical outcomes. We focused on analyzing the features of HH in pediatric patients, presenting the data as mean with standard deviation (SD) or percentages. We excluded other article types such as reviews, commentaries, and letters to the editor. Non-English language articles were also excluded. Ethical approval was not required for the preparation of this article. We analyzed the neuropathophysiological mechanisms and hypotheses proposed in the literature to explain the different clinical manifestations and variations in HH recurrence between the adult and pediatric age groups.

## 3. Results

### 3.1. The results of literature search and screening

**Figure 1:**
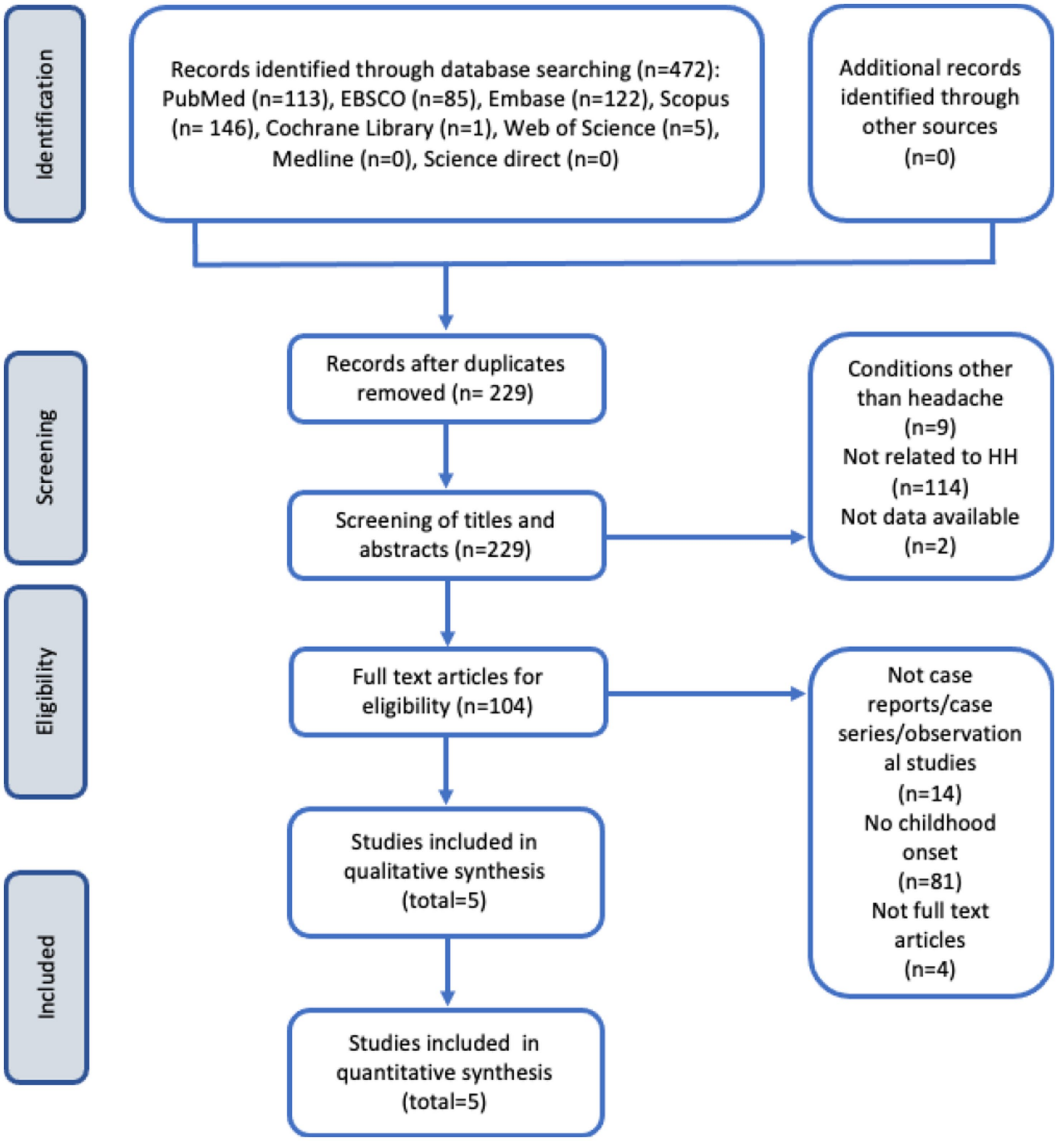
Flow diagram of study selection process according to the preferred reporting items for systematic reviews and meta-analyses (PRISMA) guidelines (13).

A total of 472 studies were initially identified from the electronic database search. After removing 243 duplicate studies, 229 studies remained for screening. During the screening process, 9 articles unrelated to headache conditions and 114 articles unrelated to HH were excluded. Additionally, 2 articles without available data were excluded, leaving 104 articles for eligibility assessment. Among these, 14 articles that did not meet the criteria of being case reports, case series, or observational studies were excluded. Furthermore, 81 articles described HH cases with an onset occurring after the age of 18 and 4 articles lacked full-text availability. Finally, a total of 5 articles met all the inclusion criteria and were included in the review (Figure 1).

### 3.2. Description of the included patients

From the 5 selected articles, 7 patients were identified who reported an onset of HH during childhood (14–18). The main characteristics and headache profiles of these patients have been summarized in Table 2. Patients #A–E have been reported in the review that specifically analyzed pediatric patients in 2015 (19). Patients #F and #G were described in separate case reports in adulthood (17, 18), but both experienced HH onset at the age of 15 with headache characteristics that are not reported to have changed over time. Four out of seven patients were females (57%). The mean age of onset for headache attacks was 10 ± 4.3 years (ranging from 3 to 15 years), and the age at publication of the case reports was 15.6 ± 13.6 years (ranging from 7 to 45 years). The diagnosis for patients #A–E was made on average 15.8 ± 25.0 months (ranging from 1 to 60 months) after the onset of the headache. The time interval from symptom onset to diagnosis was not reported for patients #F and #G. All patients experienced awakening due to pain during nocturnal sleep. Five patients (71.4%) woke up a few hours (ranging from 1 to 6 h) after falling asleep, while in two patients (#B and #D), awakening occurred during the early hours of the morning (between 2 am and 5 am). Except for patient #G (where data was not reported), the duration of the headache attacks exceeded 10–15 min, with some lasting up to 60 min and one case lasting 5 h. After experiencing relief from pain, the patients were able to go back to sleep. The mean frequency of attacks was 12 ± 2.8 days per month (ranging from 1 to 25 days per month), with the frequency for patient F being indeterminable due to the relapsing-remitting nature of the condition. Unilateral pain was reported in one case (14.3%), unilateral and bilateral pain in two cases (28.6%), and bilateral pain in 4 out of 7 patients (57.1%). The headache attacks were described as throbbing/pulsating in 3 out of 7 patients (42.8%) and as dull and stabbing in one case (14.3%). All patients reported pain ranging from moderate to severe intensity. None of the patients reported concomitant migraine features, except for occasional nausea in two patients (28.6%) and occasional photophobia in one patient (14.3%). Neurological examinations were unremarkable for all patients. Brain MRI and EEG were performed in 6 out of 7 cases, all of which showed normal results. Two patients had a positive family history of headache with one patient’s mother suffering from migraine with aura. Patient #D had thalassemia major and underwent bone marrow transplantation 6 months before the onset of HH. Their pharmacological therapy included cyclosporine, methylprednisolone, amoxicillin, fluconazole, and acyclovir (14). Patient #G reported sporadic heart palpitations, mitral valve prolapses, seasonal allergies, and obstructive sleep apnoea syndrome (OSAS) but had no other reported comorbidities. Six patients had follow-up clinical examinations for an average of 6.1 ± 4.8 months, ranging from 6 weeks to 12 months. Only two patients received acute-phase medication: patient #B found relief after taking acetaminophen approximately 30–60 min after the onset of the attack (15), while patient #F did not benefit from oral sumatriptan (100 mg), oral rizatriptan (5 mg), or oxygen inhalation. Most of the patients (57%) did not require medication, as each attack completely resolved in about 30 min. Two patients (#D and #E) received prophylactic treatment with melatonin (14). In the first patient (patient #D), an initial dose of 2 mg of melatonin at bedtime was administered. This resulted in a reduction in headache intensity (from severe to moderate) and frequency (from 25–30 attacks per month to 10–15 attacks per month). Consequently, the dosage was increased to 4 mg and over 6 months, the patient became asymptomatic (14). In the second patient (patient #E), an initial dose of 3 mg of melatonin at bedtime was given. The severity of headaches decreased from severe to moderate, and there was an immediate reduction in attack frequency from 12–15 attacks per month to 1–2 attacks per month. The patient remained headache-free during the subsequent 2 months (14). Patient #F did not report any effect in terms of frequency or severity of headache attacks after trial treatment with sodium valproate (500 mg twice daily), amitriptyline (75 mg once daily), duloxetine (60 mg twice daily), naproxen (500 mg twice daily), ibuprofen (600 mg three times daily), or propranolol (60 mg twice daily). However, an immediate response was observed when patient #F took 75 mg of indomethacin at bedtime. Indomethacin was successfully tapered off after 6 weeks, and there was no relapse during the following 3 months of follow-up (18). Patient #G, who also had co-morbid OSAS, reported remission of HH after using an oral appliance for mandibular advancement. However, the follow-up period for this patient lasted only 6 weeks (17). Overnight polysomnography was not conducted for any of the patients included in this review.

**Table 2:** Characteristics and headache profile of the seven patients with onset of hypnic headache in childhood.

Patient/headache characteristics	A	B	C	D	E	F	G
References	Grosberg et al. (16)	Scagni et al. (15)	Cerminara et al. (14)	Cerminara et al. (14)	Cerminara et al. (14)	Prakash et al. (18)	Bender (17)
Gender	Female	Female	Male	Male	Female	Male	Female
Age of attacks onset (years)	9	3	7	11	10	15	15
Age (y) of the patient at the time of publication	9	8	7	11	10	19	45
Latency till diagnosis (months)	2 months	5 years	6 months	1 month	10 months	na	na
Time of attacks	5–6 h after falling asleep	2–4 a.m.	1–3 h after falling asleep	1–2 a.m. and 4–5 a.m.	1–2 h after falling asleep	1–2 a.m., 3–4 h after falling asleep	2.5–3 h after falling asleep
Character of pain	Throbbing	Pulsating	na	Dull	Pulsating	Non-throbbing	Stabbing
Localization	Right frontal and temporal	Frontal	Fronto-temporal	Fronto-temporal	Fronto or fronto-temporal	Left frontal and temporal areas	Frontal, temporal and periocular
Laterality (uni−/bilateral)	Unilateral	Bilateral	Bilateral	Bilateral	Bilateral	Unilateral and bilateral in about 10% of attacks	Uni-and bilateral
Intensity of pain	Moderate to severe	Severe	Moderate	Moderate to severe	Moderate to severe	Moderate to severe	na
Migraineurs features (nausea, vomiting, photo-/phonophobia)	None	None	None	Occasional nausea	Occasional nausea	None	Occasional photophobia
Autonomic symptoms	None	None	None	None	None	na	Bilateral nasal congestion but no any other autonomic features
Duration of attacks (min)	30	30–60	20–30	10–20	10–30	30 min–5 h	na
Frequency of attacks (days/month)	8–12	1	2	20–25 (2–3/night)	10–15 (2–3/night)	Not evaluable: relapsing-remitting type disturbance: in each relapse, headache almost every night. Periods of relapse/remission were variable (few weeks to 6 months)	12–16 (seem to have a seasonal feature)
General/neurologic exam	Normal	Normal	Normal	Normal	Normal	Normal	Normal
Brain MRI and EEG	Normal	Normal	Normal	Normal	Normal	Normal	na
Comorbidities	None	None	None	Thalassemia major	None	None	Heart palpitations, mitral valve prolapse, seasonal allergies, OSAS
Family history of headache	Negative	Positive	Positive (mother: migraine)	Negative	Negative	na	na
Acute treatment	No	Acetaminophen with benefit	No	No	No	Oral sumatriptan, oral rizatriptan, and oxygen inhalation without benefit	na
Prophylactic therapy treatment	No	No	No	Melatonin with benefit	Melatonin with benefit	Sodium valproate, amitryptiline, duloxetine, naproxen, ibuprofen, and propranol without benefit. Indomethacin with benefit	Treated for OSAS with mandibular advancement oral appliance with benefit
Follow up time (months)	2	12	12	6	3	na	1,5 (=6 weeks)

*na, not available; OSAS, obstructive sleep apnea syndrome.

## 4. Discussion

HH is a rare form of primary headache that predominantly affects adults and can persist for years without remission of symptoms (9). Among the 348 cases included in the most recent literature review (6), only 5 cases involved children. We identified these cases from the original reports and discovered two additional patients (referred to as subjects #F and #G) reported in adults but with symptom onset occurring at 15 years of age and remaining unchanged over time. These cases suggest that the pediatric subtype of HH may persist into adulthood in certain individuals (17, 18). In this article, we examine the differences in key HH features between the two age groups. Despite the limited number of reported patients, we were able to identify some distinctions in the clinical phenotype of pediatric HH compared to adults. Additionally, we discuss the improved sensitivity of the new ICHD-3 criteria compared to the previous edition in diagnosing this type of headache. We also address the pathophysiological hypotheses, differential diagnoses, and potential treatments specific to children. The underlying reasons for the rarity, underdiagnosis, and underreporting of HH in childhood are further explored in the subsequent discussion.

### 4.1. Comparison of HH features between pediatric and adult populations

When comparing the data of the 7 patients herein described with the latest review of 343 adult patients (6), several characteristics appear to overlap. Both pediatric and adult patients experienced pain exclusively during nocturnal sleep or diurnal naps, resulting in awakening for all cases. The condition predominantly affects females, with a prevalence of 69% in adults and 57% in children, consistent with recent studies (4, 6, 8–10). Bilateral pain is more frequently observed in children (85%) compared to adults (55%), and both populations reported moderate to severe pain intensity (100% in children, 94% in adults). Neither age group exhibited migraine-associated features (57.1% in children, 62.6% in adults) or autonomic symptoms (83.3% in children, 92.4% in adults). However, several differences between the two age groups have been observed. Pediatric patients tend to have a shorter diagnosis latency (15.8 ± 25.0 months, range 1 to 60 months) compared to adults (7.6 ± 14.2 years, range 0.1–39 years). This may be attributed to the heightened concern among caregivers, leading to earlier medical consultations. The frequency of attacks is lower in children (12 ± 2.8 days/month, range 1–25 days/month) compared to adults (21.9 ± 7.6 days/month, range 3–31 days/month). In pediatric patients, attacks occur a few hours after falling asleep in 71.4% of cases, while in adults, most episodes occur between 2 and 4 a.m. (51.1%). Attack duration differs significantly, with adults experiencing longer episodes (93.6 ± 65.3 min, range 10–600 min) compared to children, whose episodes typically last up to a maximum of 60 min, except for one patient who reported pain persisting for up to 5 h. Pain quality is predominantly described as dull or pressure-like in adults (74.4%), whereas throbbing or pulsating character is more frequently reported by children (42.8%). This finding highlight that the differences in the main features of HH between adults and children outweigh the overlapping characteristics. Considering that 5.5% of adult patients had fewer than 10 episodes per month, which is the cut off considered in the diagnostic criteria, the episodic form of HH has been proposed (6), which could align with the characteristics observed in children. The clinical presentation of primary headache disorders, such as migraine, can also differ between childhood and adulthood (20). The ICHD-2 acknowledged some differences in migraine characteristics between the two age groups (11). For example, migraine pain is commonly unilateral in adults but often holocranic in children (21). Differences in the evolution of migraine pain have also been observed (21). The reasons behind these divergent clinical manifestations of certain headache disorders between pediatric and adult populations are not yet fully understood. It has been postulated that differences in myelination degree and synaptic maturation may contribute (21). However, these discrepancies are not adequately accounted for in the latest version of the diagnostic criteria, which may impede the identification of affected children.

### 4.2. Changes in the diagnostic criteria (ICHD-2 versus ICHD-3)

One of the reasons why children did not meet the criteria of the ICHD-2 was the requirement of unilateral location (22). Additionally, children did not fully satisfy criterion 3 of the ICHD-2 for diagnosing HH, which requires the headache to occur after the age of 50 years. With the revision of the ICHD-2 (11), significant changes were made to criterion “C”; the occurrence of headache was reduced from 15 to 10 days per month, and the requirement of “first occurs after the age of 50 years” was eliminated (refer to Table 1) (12). According to the ICHD-3, HH can occur at any age, including pediatric cases, although such cases are extremely rare. Furthermore, the ICHD-3 no longer states that the patient must not have more than one migraine feature (such as nausea, vomiting, and photo/phono-phobia). The criteria for “probable HH” in the ICHD-3 are even more inclusive (12). In this review, all seven cases described were diagnosed according to the ICHD-2, and four children did not fully meet the diagnostic criteria (see Table 3). In fact, they exhibited only one of the characteristics of criterion C, which is that the headache lasts for ≥15 min after waking up. As for the ICHD-3, only 2 children did not meet criterion C. However, all patients met the ICHD-3 criteria for probable HH. This data highlights the increased sensitivity of ICHD-3 in diagnosing HH in childhood compared to ICHD-2. Despite the diagnostic criteria for HH being the same for both adult and pediatric patients, certain features remain inconsistent, particularly the frequency of attacks. This study underscores the need for a separate diagnostic criterion for HH in children, emphasizing the lower frequency of attacks in this population.

**Table 3:** Diagnostic criteria for hypnic headache (HH) and probable HH according to ICHD-2 and ICHD-3.

	Patients with onset of hypnic headache in childhood and references						
	A	B	C	D	E	F	G
	Grosberg et al. (16)	Scagni et al. (15)	Cerminara et al. (14)	Cerminara et al. (14)	Cerminara et al. (14)	Prakash et al. (18)	Bender (17)
ICHD-2 for HH	- (C*)	- (C*)	- (C*)	+	- (C*)	+	+
ICHD-3 for HH	+	- (C**)	- (C**)	+	+	+	+
ICHD-3 for probable HH	+	+	+	+	+	+	+

“+” indicates that the diagnostic criteria are met. “-” indicates that the diagnostic criteria are not met, with the unmet criterion specified in parentheses. *The two conditions of criterion C from ICHD-2, “occurs more than 15 times per month” and “first occurs after the age of 50 years,” are not satisfied. **The condition of criterion C from ICHD-3, “occurring on ≥10 days/month for >3 months,” is not satisfied.

### 4.3. Pathophysiology

The intricate relationship between sleep and headache has captivated human curiosity for centuries, yet it remains poorly understood. Sleep is associated with certain headache syndromes, and headaches can disrupt sleep hygiene, while sleep disturbances can trigger or exacerbate the frequency and severity of headache attacks and their comorbidities (23–26). Similar to other headache types, the exact pathophysiological mechanisms of HH in childhood have not yet been fully elucidated. It has been proposed that HH may be a chronobiological disorder (2). Many patients consistently experience headache attacks at the same time during the night, commonly known as “alarm-clock headache.” Hypothalamic dysfunction and dysregulation of serotonin and melatonin have also been hypothesized as contributing factors to the disease (10, 27). The suprachiasmatic nucleus (SCN) of the hypothalamus, which plays a crucial role in the endogenous circadian rhythm, is considered a potential region involved in the onset of HH (28). The number of cells in the SCN significantly decreases with age (10, 29). The SCN projects efferent and receives afferent signals from the brainstem periaqueductal grey, locus coeruleus, and raphe nuclei, which are important brainstem structures involved in pain modulation and sleep (10, 29–31). With advancing age, the activity of the hypothalamic-pineal axis and SCN decreases, resulting in reduced melatonin production, which can be completely absent after the age of 60 years (10, 29, 32, 33). Dysregulation of melatonin synthesis is a well-known documented factor in headache development (2, 31, 34, 35). These age-related changes may explain the significantly higher incidence of HH attacks in adult patients. The hypothesis of posterior hypothalamic involvement in the pathophysiology of HH is supported by findings of decreased grey matter volume in this brain region in HH patients compared to age-and gender-matched healthy controls (36). Headaches associated with sleep can occur during sleep, after sleep, and during different sleep stages (37). It was initially suggested that HH was associated with rapid eye movement (REM) sleep, suggesting it was a REM-sleep disorder (38–41). However, subsequent studies contradicted this notion and demonstrated that HH attacks predominantly occurred during non-rapid eye movement (NREM) sleep (42–46). Further analysis did not reveal a clear REM or NREM subtype of HH, as both types of headache attacks could be observed in the same patient on the same night (42). Instead, HH attacks may be linked to changes in sleep microstructure. In a single patient treated with amitriptyline, a slight increase in cyclic alternating pattern (CAP) rate, from 40 to 46%, was observed despite the improvement of nocturnal symptoms and overall NREM sleep parameters (47). The authors concluded that studying the macro and microstructure of sleep could be valuable in elucidating the pathological mechanisms of HH, although no further studies have pursued this line of research. Additionally, similar to other headache forms, mood fluctuations, changes in daylight exposure, environmental temperature, and lifestyle factors may trigger the recurrence of HH (48).

### 4.4. Differential diagnosis

The main differential diagnosis for HH encompasses secondary headaches and other primary headache disorders (12, 49). In the case of secondary headaches, it is crucial to rule out other potential conditions that can occur during sleep and lead to patient awakening, with specific attention to hypoglycemia (50), medication overuse (12, 51, 52), and sleep apnoea (53). Overnight polysomnography may be necessary in selected cases to exclude OSAS and other sleep disorders. However, the presence of OSAS does not necessarily exclude an HH diagnosis (12, 17). Additionally, intracranial vascular pathologies (54–56) and brain tumours must be considered and ruled out (57–60). Brain MRI is required to investigate any structural abnormalities, such as tumours and it may also reveal a reduction in grey matter volume in the hypothalamus (36). Nocturnal hypertension has also been identified as a potential cause of HH (61–63), and 24 h blood pressure monitoring is recommended in patients with cardiovascular risk factors to assess nocturnal hypertension. Primary headache disorders that tend to occur during sleep, such as migraine, cluster headaches, paroxysmal hemicrania, cervicogenic headache, and short-lasting, unilateral, neuralgiform headache attacks with conjunctival injection and tearing (SUNCT), should also be investigated before reaching an HH diagnosis (12, 32). After that other primary headaches and any secondary or organic causes of pain-induced awakenings are ruled out, the diagnosis of HH is primarily based on clinical assessment and adherence to the ICHD-3 diagnostic criteria.

### 4.5. Therapeutic options in children

Recommendations for the acute and prophylactic treatment of HH attacks in children are scarce and primarily based on individual case reports, case series, and literature reviews (6, 14–19). In adults, prophylactic treatment has shown better outcomes (6). Two patients included in this review (patients #D and #E) benefited from melatonin therapy and achieved prompt relief from headaches. These results may provide empirical evidence of the involvement of melatonin and the circadian rhythm system in HH pathogenesis. However, these findings differ from those reported in the adult population, where the response rate to melatonin is approximately 50% (6, 64, 65). Melatonin, a chronobiotic hormone primarily secreted by the pineal gland during the night, has demonstrated therapeutic efficacy in certain forms of headache (66, 67). Patient #F showed improvement with indomethacin treatment. Indomethacin has better blood–brain barrier penetration compared to other nonsteroidal anti-inflammatory drugs, such as naproxen and ibuprofen, and specifically inhibits nitric oxide-induced dural vasodilation (68). This may explain the greater benefit of indomethacin in HH and other headache types (6, 68–70). In patient #G, mandibular advancement with an oral appliance for OSAS resulted in complete control of HH symptoms; however, only a 6 week follow-up period is available for this patient. Although OSAS has been frequently associated with HH, prevalence studies have not confirmed a higher prevalence of OSAS in HH patients compared to unaffected individuals (41). Previous reports on the use of positive airway pressure in HH patients have yielded inconclusive results (10, 39, 40, 70). In one case series, polysomnography recordings in HH patients did not reveal any episodes of OSAS (39). One patient showed improvement with continuous positive airway pressure therapy and supplemental oxygen, while three patients did not (40). Currently, treatment recommendations for HH in children are based solely on anecdotal case reports and uncontrolled trials in small case series, emphasizing the need for prospective trials comparing different prophylactic treatment strategies against placebo. Since HH attacks in pediatric patients generally last less than 60 min, episodes often go untreated. Only patient #B experienced benefit from acetaminophen, while patient #F tested various medications (oral sumatriptan, oral rizatriptan, and oxygen inhalation) without encouraging results. Similar to experiences with adult patients, lithium and caffeine may prove effective in the pediatric population, but larger placebo-controlled studies are required to validate this hypothesis (6).

## 5. Conclusion

After the revision of ICHD-2 (11), changes in ICHD-3 have improved the accuracy of HH diagnosis. Although the criteria are considered more sensitive, as demonstrated by our systematic review and previous articles (9), and the age limit for diagnosis has been removed, no new cases of pediatric HH have been published since 2012 (17). This may be due to the limited knowledge of detailed clinical features of HH in childhood. Comparing headache features in adults and children, it is conceivable that different developmental age-related stages may be associated with distinct clinical presentations (20, 21, 71). The scientific understanding of HH clinical and diagnostic differences between pediatric and adult populations will likely become clearer in the future. It raises the question of whether it would be more appropriate to expand the ICHD-3 diagnostic criteria for HH (which may lead to overdiagnosis of forms that are not entirely classifiable) or to establish separate classification criteria for adults and the pediatric age group. This study emphasizes the need for separate diagnostic criteria for HH, highlighting differences in pain quality (throbbing/pulsating in children vs. dull/pressure in adults) and lower frequency and duration of attacks in children. This could enhance the sensitivity of diagnostic criteria in identifying affected children and managing misdiagnosed cases. Other primary headaches and secondary causes must be ruled out during HH diagnosis. Although the sample size is very small and heterogeneous due to the rarity of this condition and underdiagnosis resulting from criteria that do not consider distinct pediatric characteristics compared to adults, prophylactic therapy with melatonin appears to be the most promising strategy in the pediatric age group. Current research has several limitations: (1) the small sample size due to the rarity of the condition and underdiagnosis, (2) lack of homogeneous descriptions of pediatric patients across different articles, and (3) absence of placebo-controlled studies to verify the efficacy of acute and prophylactic drugs in the pediatric age group, limiting the generalizability of results. Future research directions should focus on larger case studies to gather more information on pediatric HH features and improve the management and quality of life of young patients.

## Data availability statement

The original contributions presented in the study are included in the article, further inquiries can be directed to the corresponding author.

## Author contributions

AF: Data curation, Investigation, Methodology, Writing - original draft, Conceptualization, Writing - review & editing. MV: Data curation, Formal analysis, Investigation, Methodology, Writing – original draft. CF: Data curation, Formal analysis, Investigation, Methodology, Writing - original draft. GN: Data curation, Formal analysis, Investigation, Methodology, Writing – original draft. ME: Data curation, Formal analysis, Investigation, Methodology, Writing – original draft. TF: Data curation, Formal analysis, Investigation, Methodology, Writing – original draft. AO: Data curation, Investigation, Methodology, Writing – original draft, Formal analysis. MP: Data curation, Formal analysis, Investigation, Methodology, Writing – original draft. GT: Data curation, Formal analysis, Investigation, Methodology, Writing – original draft. UR: Conceptualization, Supervision, Writing – review & editing. PS: Conceptualization, Supervision, Writing – review & editing. PP: Conceptualization, Supervision, Writing – review & editing.

## Funding

This work was supported by #NEXTGENERATIONEU (NGEU) and funded by the Ministry of University and Research (MUR), National Recovery and Resilience Plan (NRRP), project MNESYS (PE0000006)-A Multiscale Integrated Approach to the Study of the Nervous System in Health and Disease (DN. 1553 11.10.2022). IRCCS ‘G. Gaslini’ is a member of ERN-Epicare. This work was also supported by the Italian Ministry of Health, RICERCA CORRENTE 2023.

## Conflict of interest

The authors declare that the research was conducted in the absence of any commercial or financial relationships that could be construed as a potential conflict of interest.

## Publisher’s note

All claims expressed in this article are solely those of the authors and do not necessarily represent those of their affiliated organizations, or those of the publisher, the editors and the reviewers. Any product that may be evaluated in this article, or claim that may be made by its manufacturer, is not guaranteed or endorsed by the publisher.
